# Patientenorientierte Optimierung der Versorgungsqualität in der ambulanten spezialfachärztlichen Versorgung (ASV) in einem tertiären rheumatologischen Zentrum: Eine qualitative Studie

**DOI:** 10.1007/s00393-024-01520-z

**Published:** 2024-05-28

**Authors:** Vlora Ibishi, Uta Kiltz, Styliani Tsiami, Michael Wessels, Xenofon Baraliakos

**Affiliations:** 1 Studiengang: Gesundheit und Diversity in der Arbeit, Hochschule für Gesundheit Bochum, Bochum, Deutschland; 2 Ruhr-Universität Bochum, Bochum, Deutschland; 3 Rheumazentrum Ruhrgebiet, Claudiusstr. 45, 44649 Herne, Deutschland; 4 Department of Community Health, Hochschule für Gesundheit, Bochum, Deutschland

**Keywords:** Versorgungsqualität, Ambulante spezialärztliche Versorgung, Patientenorientierung, Qualitative Forschung, Entzündlich-rheumatische Erkrankungen, Quality of care, Outpatient specialist care, Patient orientation, Qualitative research, Inflammatory rheumatic diseases

## Abstract

**Hintergrund:**

Die Anpassung von Strukturen und Prozessen der Behandlungsabläufe kann zur Steigerung der Patientenzufriedenheit beitragen und steht im Fokus einer patientenorientierten Qualitätssicherung.

**Ziel:**

Es erfolgte die Identifizierung der Patientenzufriedenheit sowie der Bedürfnisse, Erwartungen und Präferenzen hinsichtlich der Versorgung und daraus ableitend die Formulierung von Handlungsempfehlungen zur Optimierung der Versorgungsqualität eines großen tertiären rheumatologischen Zentrums.

**Material und Methode:**

Im Rahmen eines qualitativen Forschungsansatzes wurden halbstrukturierte Patienteninterviews und ein Fokusgruppeninterview bestehend aus Ärzt:innen in rheumatologischer Weiterbildung (ÄiWB) in der ambulanten spezialfachärztlichen Versorgung (ASV) durchgeführt. Es wurden die Qualitätsdimensionen nach Donabedian erfasst. Das Datenmaterial wurde anhand der inhaltlich-strukturierenden qualitativen Inhaltsanalyse (QIA) nach Kuckartz mit der Auswertungssoftware MAXQDA ausgewertet und analysiert.

**Ergebnisse:**

Mittels 12 Patienteninterviews und einer Fokusgruppe aus 3 ÄiWB wurden auf Grundlage der Struktur‑, Prozess- und Ergebnisqualität Handlungsempfehlungen zur Optimierung der Versorgungsqualität abgeleitet. Es erwies sich Optimierungsbedarf im Bereich des Personalmanagements, der internen Praxisabläufe, der Praxisausstattung und der Behandlungsabläufe in der ASV-Ambulanz.

**Schlussfolgerungen:**

Die Ergebnisse aus den Patienteninterviews und der Fokusgruppe zeigten die Aspekte mit Optimierungsbedarf auf. Die Methodik und Ergebnisse dieser Studie können als Anhaltspunkt für Analysen anderer rheumatologischer Kliniken dienen, um im Rahmen des patientenorientierten Qualitätsmanagements und der kontinuierlichen Weiterentwicklung die Versorgungsqualität zu verbessern.

## Abstract (en)

**Background:**

The adaptation of structures and processes in treatment procedures can contribute to increasing patient satisfaction and is the focus of patient-oriented quality assurance.

**Objective:**

To identify patient satisfaction as well as needs, expectations and preferences with respect to care and, based on this, to formulate recommendations for action to optimize the quality of care at a large tertiary rheumatology center.

**Material and methods:**

As part of a qualitative research approach, semi-structured patient interviews and a focus group interview consisting of physicians in rheumatology training in outpatient specialist care were conducted. The quality dimensions of Donabedian were recorded. The data material was evaluated and analyzed using the content-structuring qualitative content analysis according to Kuckartz with the MAXQDA evaluation software.

**Results:**

Using 12 patient interviews and a focus group of 3 future rheumatologists, recommendations for action to optimize the quality of care were derived on the basis of the structural, process and outcome quality. There was a need for optimization in the areas of personnel management, internal practice processes, practice equipment and treatment processes in the outpatient clinic.

**Conclusion:**

The results from the patient interviews and the focus group revealed the aspects in need of optimization. The methodology and results of this study can serve as a reference point for analyses of other rheumatology clinics in order to improve the quality of care within the framework of patient-oriented quality management and continuous further development.

## Hintergrund

Die Sicherstellung einer bestmöglichen Versorgung stellt einen wesentlichen Faktor im Versorgungssystem dar. Die Versorgungsqualität ist ein Bestandteil der Versorgungsforschung und zielt darauf ab, die Krankheits- und Gesundheitsversorgung kontinuierlich zu verbessern und die Qualität der Versorgung zu erhalten. Dabei werden die Strukturqualität (SQ), Prozessqualität (PQ) und Ergebnisqualität (EQ) berücksichtigt [1].

Die ambulante spezialfachärztliche Versorgung (ASV) setzt den Fokus auf die Diagnostik und Behandlung von Patient:innen mit chronischen und/oder komplexen Erkrankungen, oftmals auch seltenen Erkrankungen, die sich in ihren Krankheitsverläufen besonders schwierig gestalten und somit eine hoch spezialisierte Versorgung erfordern. Die Versorgungsleistungen werden durch ein aus verschiedenen Fachrichtungen qualifiziertes interdisziplinäres Fachärzt:innen-Team (ASV-Team) erbracht. Durch die ASV kann die fachärztliche Versorgung im deutschen Gesundheitssystem im Hinblick auf seltene, chronische und komplexe Erkrankungen verbessert werden [2].

Das Rheumazentrum Ruhrgebiet (RZR) ist das größte hoch spezialisierte tertiäre rheumatologische Zentrum deutschlandweit, welches besonders Patient:innen mit rheumatischen und muskuloskeletalen Gelenkerkrankungen sowie immunologischen Erkrankungen eine optimale stationäre, ambulante, diagnostische und therapeutische Versorgung anbietet [3, 4]. Darüber hinaus ist das RZR besonders durch sein umfassendes, flexibles und an die Patient:innen angepasstes Versorgungskonzept gekennzeichnet. Das Versorgungskonzept besteht aus einer engen Verzahnung der stationären und ambulanten Versorgung. Die ambulante Versorgung wird im Rahmen einer ASV erbracht, welche durch ein spezialisiertes und qualifiziertes ASV-Team mit Rheumatolog:innen und weiteren Fachrichtungen eine Versorgung für Patient:innen mit schweren und komplexen rheumatologischen Erkrankungen erbringt [5].

Im Rahmen der rheumatologischen Versorgung stellt die Anpassung des Versorgungskonzeptes an die Bedürfnisse, Erwartungen sowie Präferenzen der Patient:innen einen relevanten Aspekt dar. Die Relevanz der individuellen Versorgungsbedürfnisse zeigte sich bereits in Studien, die im Rahmen dieser die Zufriedenheit der Patient:innen sowie den Versorgungsbedarf und die Erwartungen an die rheumatologische ambulante Versorgung erhoben. Zusammenfassend zeigten die Studien von Ward et al. (2007) und Kjeken et al. (2006), dass Patient:innen einen Versorgungsbedarf erlebten und somit eine Unzufriedenheit mit der Versorgung aufwiesen. Demnach listeten sie spezifische Bedürfnisse auf, zu denen die Patientenaufklärung (Information über Diagnose, Medikamente), die Unterstützung bei der Ausführung alltäglicher Aktivitäten sowie die Schmerzbehandlung gehören [6, 7]. Eine Studie von Mercieca (2013) schaffte ein besseres Verständnis für die Bedürfnisse und Erwartungen der Patient:innen in einer rheumatologischen Ambulanz, dessen Erkenntnisse ergaben, dass Patient:innen mit der Wartezeit, den Termineinhaltungen, der räumlichen Ausstattung und dem unorganisierten Wartebereich unzufrieden waren [8].

Die Qualität der täglichen medizinischen Versorgung basiert auf den intern abgesprochenen Strukturen und Prozessen, die den Behandlungserfolg garantieren sollen. Diese 3 Aspekte sind an die Patientenbedürfnisse, -erwartungen und -präferenzen angepasst. Darüber hinaus liegt der Qualitätssicherung der sog. strategische Dreiklang zugrunde, in dessen Rahmen das RZR kontinuierlich an der Strukturqualität und Prozessqualität arbeitet, um die Verbesserung der Ergebnisqualität zu gewährleisten. Das RZR nimmt an Qualitätssicherungsmaßnahmen, z. B. am KOBRA-Projekt durch den Verband für rheumatologische Akutkliniken (VRA), teil [9]. Ziel des langjährigen Projektes ist die Qualitätsmessung der akut-stationären Versorgung. Hierzu erhält das RZR laufend das Gütesiegel für seine kontinuierliche hochqualitative Versorgung von Patient:innen [10].

Das Ziel dieser Arbeit ist es, die Patientenzufriedenheit mit der bisher in Anspruch genommenen Versorgung sowie die patientenorientierten Bedürfnisse, Erwartungen und Präferenzen hinsichtlich der Versorgung im Rahmen des ASV-Konzepts zu identifizieren und anschließend auf Basis der Qualitätsdimensionen nach Donabedian Handlungsempfehlungen zur Optimierung der Versorgungsqualität abzuleiten. Die Erkenntnisse dieses Forschungsvorhabens sollen darüber hinaus das unternehmensinterne QM unterstützen, um eine bestmögliche Versorgungsqualität in der ASV-Ambulanz zu erlangen.

## Material und Methoden

### Studiendesign

Das Forschungsvorhaben basiert auf einem qualitativen Forschungsansatz, der sich primär mit der Erhebung und Interpretation von Textmaterialen, wie z. B. Interviewtranskripten, befasst. Im Gegensatz zur qualitativen Forschung dominiert im quantitativen Forschungsansatz die schriftliche Befragungsmethode, die mittels Fragebögen durchgeführt wird und eher eine alltagsnahe Erhebungsmethode darstellt. Im Rahmen quantitativer Erhebungsmethoden wird ein größerer Umfang an Daten erhoben, die daraufhin statistisch ausgewertet werden. Im Rahmen dieser Arbeit wurde auf diesen Forschungsansatz verzichtet und auf den qualitativen Forschungsansatz zurückgegriffen, der anhand seiner Methoden eine zwischenmenschliche Kommunikation und Interaktion zwischen der durchführenden Person und den Teilnehmenden ermöglicht, Aspekte des subjektiven Empfindens und Erlebens der Patient:innen zugänglich macht und es ermöglicht, direkte Erwartungen zu erfassen [11].

**Abb. 1:**
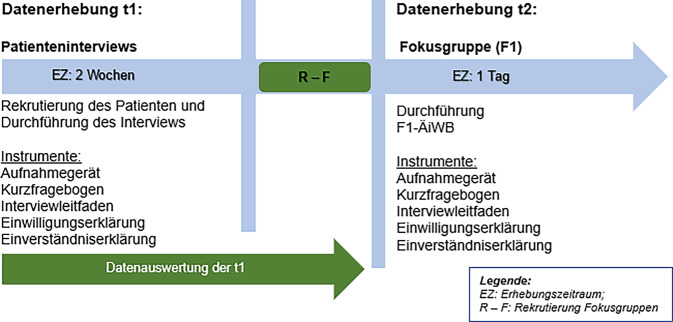
Darstellung des Studiendesigns

Im Rahmen der Forschung wurden in einem qualitativen Forschungsansatz Patienteninterviews (t1) und ein Fokusgruppeninterview mit ÄiWB (t2) durchgeführt. Die Ergebnisse der Patienteninterviews zu t1 dienten zur Information der ÄiWB in der anschließend stattfindenden Fokusgruppe (FG) t2 (Abb. 1). In t1 wurden die Zufriedenheit, die subjektiven Bedürfnisse, Erwartungen und Präferenzen der Patient:innen mit der medizinischen Versorgung in der ASV-Ambulanz erhoben. Im Rahmen der t2-Erhebung wurden die Ergebnisse der Patienteninterviews aus der t1-Erhebung den ÄiWB der Fokusgruppe vorgestellt. In der t2-Erhebung wurde die Sicht der teilnehmenden ÄiWB auf die Versorgungsqualität erhoben. Mit der t2-Erhebung sollen eine weitere Perspektive auf die Patientenergebnisse sowie die Sicht der ÄiWB auf die ablaufenden Prozesse in der ASV-Ambulanz dargestellt werden. Die Erkenntnisse aus t1 und t2 ermöglichten im letzten Schritt die Ableitung von Handlungsempfehlungen (HE) zur Optimierung der Versorgungsqualität in der ASV-Ambulanz.

### Stichprobe

#### Qualitatives Interview mit Patient:innen

Patient:innen konnten an der t1-Erhebung teilnehmen, sofern sie folgende Einschlusskriterien erfüllten: erwachsene Patient:innen mit gesicherten entzündlichen rheumatischen Erkrankungen, die seit über 2 Jahren Patient:in der ASV-Ambulanz des RZR sind. Ausgeschlossen waren Drittpersonen (z. B. Dolmetscher:in) und Notfallpatient:innen. Die Rekrutierung erfolgte durch direkte mündliche Ansprache während der Termine in der ASV-Ambulanz ohne weitere Selektion der Patient:innen. Das qualitative Interview wurde nach erfolgreicher Rekrutierung durchgeführt. Die Erhebung patientenbezogener Daten, u. a. Name, Alter, Geschlecht und der Behandlungszeitraum in der ASV-Ambulanz, erfolgte anhand eines Kurzfragebogens.

#### Fokusgruppe – ÄiWB

In die FG wurden ÄiWB eingeschlossen, die bereits über 6 Monate Einblicke und Erfahrungen in der ASV-Ambulanz gesammelt haben und die Versorgungsstruktur und Versorgungs- sowie Ambulanzabläufe kannten. Die Rekrutierung der FG-Stichprobe erfolgte mündlich bei einem Gespräch mit einer teilnehmenden Rheumatologin. Anhand eines Kurzfragebogens wurden relevante personenbezogene Daten, u. a. Name, Alter, Geschlecht und Berufsbezeichnung, erhoben.

### Erhebungsinstrument

#### Patienteninterview (t1).

Sowohl für die Patienteninterviews als auch für die Fokusgruppe wurde ein halbstrukturierter Interviewleitfaden entwickelt. Der Leitfaden für die Patienteninterviews beinhaltet Fragen zur (I) Zufriedenheit mit der Versorgung in der ASV-Ambulanz, zu (II) Bedürfnissen, Erwartungen und Präferenzen hinsichtlich der Versorgung ihrer Erkrankung seitens der ASV-Ambulanz und Fragen zum (III) subjektiv empfundenen Optimierungsbedarf und möglichen HE zur Optimierung und Deckung des Bedarfs.

Das erste Patienteninterview diente als Testdurchlauf (Pre-Test). Das Ergebnis wurde nicht in die Auswertung miteinbezogen. Beim Testdurchlauf lag der Fokus auf der Durchführbarkeit des Interviews, der Verständlichkeit und der Beantwortbarkeit der Fragen.

Das Forschungsvorhaben sieht die Durchführung von 10 bis 15 Interviews vor. Anhand dieser Datenerhebung wird für das anvisierte Forschungsziel vorerst eine ausreichende Datensättigung erwartet, die den Gewinn erster Handlungsansätze zur Optimierung der Versorgungsqualität in der ASV-Ambulanz ermöglichen soll.

#### Fokusgruppeninterview (t2).

Der Leitfaden für das Fokusgruppeninterview beinhaltete die Ergebnisse der Patienteninterviews, die im Rahmen der t2-Erhebung präsentiert wurden. Die Ergebnispräsentation erfolgte entlang der Kategorien (I) Patientenzufriedenheit, (II) Erwartungen, Bedürfnisse und Präferenzen und (III) Handlungsempfehlungen der Patient:innen zur Optimierung der ASV-Ambulanz. Weitere Moderationsfragen dienten zur Unterstützung und Steuerung der Diskussions- und Reflexionsrunde.

### Datenauswertung

Für die Auswertung des Datenmaterials aus t1 und t2 wurde eine qualitative Inhaltsanalyse (QIA) mit dem Fokus auf die inhaltlich-strukturierende QIA nach Kuckartz durchgeführt [12]. Die Bildung des Kategoriensystems und die darauffolgende Auswertung gelangen mittels der MAXQDA-Software. Im Rahmen der Auswertung dienten die 3 Qualitätsdimensionen Strukturqualität, Prozessqualität und Ergebnisqualität nach Donabedian [13] als Basis für die Ableitung von HE zur Optimierung der Versorgungsstrukturen und -prozesse in der ASV mit Blick auf eine mögliche kontinuierliche Verbesserung der Versorgungsqualität.

### Formulierung der Handlungsempfehlung

Die Formulierung der HE erfolgte in 3 Phasen (Tab. 1). Die HE basieren auf den Erhebungen t1 und t2 und wurden von der Forschungsdurchführenden VI formuliert.

**Tab. 1:** Phasen der Entwicklung der Handlungsempfehlungen

Phase	
*1*	*Teil I:* Durchführung der t1-Erhebung: Patienteninterviews *Teil II:* Auswertung und Identifikation der Kategorien
*2*	*Teil I:* Durchführung der t2-Erhebung: Fokusgruppeninterview *Teil II:* Auswertung und Identifikation der Kategorien
*3*	Formulierung von Handlungsempfehlungen ableitend aus der t1- und t2-Erhebung

## Ergebnisse

### Auswahl und Charakteristika der Patientenstichprobe

**Abb. 2:**
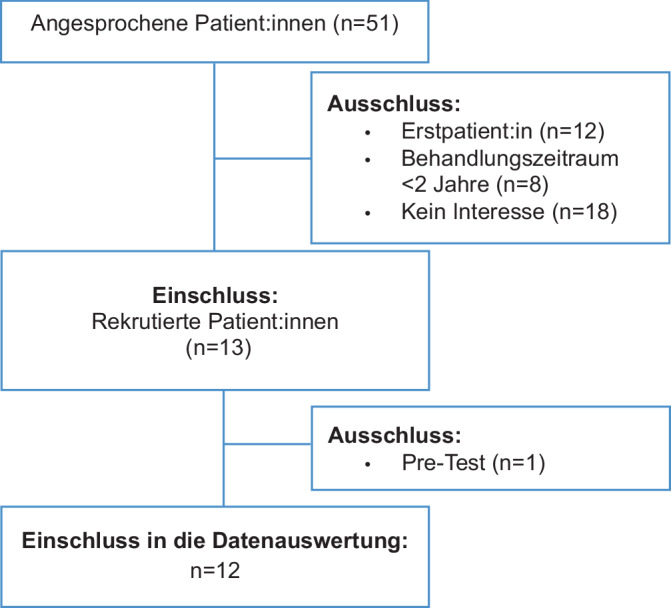
Flussdiagramm des Auswahlverfahrens: Einschluss und Ausschluss der Patientenstichprobe

Die Erhebung t1 erfolgte im Mai 2023. Von insgesamt 51 angesprochenen Patient:innen wurden 13 Patient:innen erfolgreich rekrutiert und zu dem Interview eingeladen (Abb. 2).

Das qualitative Sample besteht aus 12 Patient:innen. Die Gruppe der befragten Patient:innen setzte sich zusammen aus 4 männlichen und 8 weiblichen Patient:innen (Tab. 2). Das Durchschnittsalter lag bei 64 Jahren, wobei die Altersspanne der Patient:innen von 53 bis 77 Jahren reichte.

**Tab. 2:** Patientendaten

Patient	Geschlecht	Alter (zum Zeitpunkt der Datenerhebung)	Behandlungszeitraum in der ASV-Ambulanz
*B01*^*a*^	Männlich	37	2 Jahre
*B02*	Männlich	77	3 Jahre
*B03*	Weiblich	67	2 Jahre
*B04*	Weiblich	71	2 Jahre
*B05*	Weiblich	53	5 Jahre
*B06*	Weiblich	72	2 Jahre
*B07*	Weiblich	64	4 Jahre
*B08*	Männlich	71	4 Jahre
*B09*	Weiblich	61	11 Jahre
*B10*	Männlich	55	6 Jahre
*B11*	Weiblich	70	4 Jahre
*B12*	Weiblich	54	3 Jahre
*B13*	Männlich	63	2 Jahre

^a^Patient B01 wurde in die Auswertung der qualitativen Interviews nicht miteinbezogen

### Auswahl und Charakteristika der Fokusgruppe – ÄiWB

Die Erhebung t2 erfolgte im August 2023. Insgesamt konnten 4 ÄiWB für das FG-Interview rekrutiert werden (Tab. 3). An dem FG-Interview nahmen letztlich 3 ÄiWB teil, da eine Teilnehmende krankheitsbedingt ausgefallen ist. Die FG bestand aus 2 weiblichen ÄiWB und einem männlichen AiWB. Alle Teilnehmenden befanden sich im 6. Weiterbildungsjahr. Das durchschnittliche Alter der teilnehmenden ÄiWB lag hier bei 42 Jahren.

**Tab. 3:** Daten der FG-Teilnehmenden

Nr.	Teilnehmende	Geschlecht	Alter	Berufsbezeichnung
*1*	T1	Männlich	32	Arzt in Weiterbildung für innere Medizin und Rheumatologie (6. Weiterbildungsjahr)
*2*	T2	Weiblich	36	Arzt in Weiterbildung für innere Medizin und Rheumatologie (6. Weiterbildungsjahr)
*3*	T3	Weiblich	60	Arzt in Weiterbildung für innere Medizin und Rheumatologie (6. Weiterbildungsjahr)
*4*	*Vierte FG-Teilnehmerin ist krankheitsbedingt ausgefallen*			

## Ergebnisse der Patienteninterviews (t1)

### Patientenzufriedenheit

Im Rahmen der qualitativen Inhaltsanalyse wurden 4 Kategorien ermittelt, mit denen die Patient:innen hinsichtlich der medizinischen Versorgung in der ASV-Ambulanz zufrieden waren.

1. **Versorgung. **Die Hälfte der Patientenstichprobe (*n* = 6) äußerte ihre Zufriedenheit mit der Versorgung in der ASV-Ambulanz. Hierbei bezogen sie sich auf eine allumfassende Versorgung, die sich sowohl auf vielfältige diagnostische Möglichkeiten als auch den direkten Zugang zu therapeutischen Möglichkeiten bezog.
2. **Ärztliche Betreuung während Behandlung.** Fünf Patient:innen kommentierten die wahrgenommene ärztliche Betreuung im positiven Sinne. Sie empfanden die Ärzt:innen als professionell, entschlussfreudig, kooperativ und bemüht, die Patient:innen zufriedenzustellen. Patient:innen äußerten ihre Begeisterung über die ärztlichen Kompetenzen, die ihnen viel Vertrauen und Sicherheit in der Behandlung geben.
3. **Medizinische Behandlungsleistungen.** Darüber hinaus äußerten die Befragten ihre Zufriedenheit mit den medizinischen Behandlungsleistungen in der ASV-Ambulanz:
  *Laboruntersuchungen*
    Die meisten Patient:innen äußerten ihre Zufriedenheit mit dem Prozess der Blutentnahme, die reibungslos, problemlos und professionell abläuft.
  *Infusionen*
    Die Zufriedenheit mit den Infusionen konnte bei 2 Patient:innen erhoben werden. Die anderen 10 Patient:innen erhielten keine Infusionstherapien. Die Infusionspatient:innen äußerten ihre Zufriedenheit mit dem organisierten Infusionsablauf und dem verantwortungsbewussten Handeln der Pflegefachkräfte.
  *Therapeutische Unterstützung und Therapieerfolg*
    Der Großteil der Patient:innen (*n* = 7) äußerte sich zufrieden mit den ärztlichen Ratschlägen und der Therapieempfehlung. Die Durchführung der Therapien hat die körperliche Funktionsfähigkeit, Beweglichkeit und gesundheitsbezogenen Lebensqualität verbessert.
4. **Erreichbarkeit der Ambulanz. **Die telefonische Erreichbarkeit und die Möglichkeit, als Notfallpatient:in vorstellig versorgt zu werden, stellt für die Patient:innen einen Sicherheitsfaktor für die Versorgung ihrer Erkrankung dar.

### Patienten*un*zufriedenheit

Zu folgenden Aspekten äußerten sich die Patient:innen unzufrieden:

#### Arztwechsel.

Die Arztwechsel in der individuellen Versorgung in der ASV-Ambulanz wurde von einem Teil der befragten Patient:innen (*n* = 5) als nachteilig bemerkt. Die Patient:innen wünschen eine:n feste:n Rheumatolog:in, der sie individuell betreut. Die Versorgung durch eine:n Rheumatolog:in und die Schaffung einer Vertrauensbasis sind für Patient:innen ein wichtiger Faktor in der Krankheitsversorgung und würden deren Sicherheit erhöhen.

#### Sprachbarriere.

Vier Patient:innen äußerten sich kritisch zu der aus ihrer Sicht mangelnden deutschsprachigen Kompetenzen der behandelnden Ärzt:innen. Die Patient:innen berichteten von Verständnisproblemen und Kommunikationsschwierigkeiten, die zu Verwirrungen und zur unvollständigen Patientenaufklärung führen können. Eine Patientin sah in der Sprachbarriere eine Ursache für verlängerte Wartezeiten. Für 2 Patient:innen zeigte sich die Sprachbarriere jedoch als minimales Problem, da sie keine Schwierigkeiten beim Verstehen von sprachlichen Färbungen haben.

#### Digitales Aufruf- und Nummernsystem.

Das zeitlich wechselnde und unstrukturierte Anmelde- und Aufrufverfahren des automatisierten Nummernsystems führte bei vielen Patient:innen zu Unsicherheiten. Hierzu berichteten sie von verschiedenen Sichtweisen bezüglich des Anmelde- und Nummernsystems. Die Patient:innen berichteten von einer unstrukturierten Systematik bei dem Aufruf zur Laboruntersuchung mit dem vorhandenen Aufrufsystem. Aufgrund der unstrukturierten Reihenfolge im Wartebereich entstanden Unsicherheiten bei den wartenden Patient:innen. Die Unstrukturiertheit führte zu Missverständnissen zwischen wartenden Patient:innen.

#### Technische Funktionsfähigkeit.

Das Aufrufen der Patient:innen zum Behandlungsraum erfolgte über Monitore, die im Wartebereich vorhanden waren. Ein Patient berichtete, dass die Monitore vereinzelt nicht funktioniert hätten und daher das Nummernsystem nicht sichtbar war.

#### Apparative Ausstattung.

Von einer Patientin wurde angemerkt, dass das RZR nicht über ein offenes MRT verfügen würde. Dies sei insbesondere für Patient:innen mit ankylosierender Spondylitis hinderlich, da aufgrund der körperlichen Deformität längeres Verweilen in der MRT-Röhre nicht möglich sei.

#### Räumliche Orientierung.

Die räumliche Orientierung wurde von einer Patientin als unübersichtlich angemerkt. Zudem merkte sie an, diese Unsicherheit bei weiteren Patient*innen bemerkt zu haben.

#### Wartezeiten.

Die Wartezeiten in der ASV-Ambulanz waren laut der Aussage einer Patientin „auf Kosten der Patient:innen“. Sie berichteten von mehreren verbrachten Stunden trotz eines vereinbarten Termins. Im Durchschnitt verbrachten sie insgesamt 1–3 h in der ASV-Ambulanz. Darüber hinaus berichtete eine Patientin davon, dass der Klinikbesuch bedingt durch die Wartezeit mit ihrer Arbeitstätigkeit kollidiert. Diese Problematik erkannten sie dem Personalmangel zu. Zwei Patient:innen nahmen bezüglich der Wartezeit hingegen eine Verbesserung über die Zeit, in der sie im RZR in Behandlung sind, wahr.

#### Terminmanagement.

Ein Patient kommentierte die geänderte Terminierung der regulären Kontrolluntersuchungen, die bei Patient:innen mit stabiler Krankheitsaktivität von einer quartalsweisen auf eine halbjährliche Arztvisite verlängert wurde. Diesen Aspekt schrieb er dem Personalmangel zu.

#### Rezeptmanagement.

Für eingeschränkte und immobile Patient:innen war die persönliche Abholung der Rezepte als schwierig umsetzbar angegeben.

#### Medikamentenanwendung.

Eine Patientin berichtete, dass die Anwendung ihrer Medikamente im Therapieverlauf zeitlich nicht optimal verlief. Die Medikamentengabe erfolgte zu einem späteren Zeitpunkt, hätte jedoch früher erfolgen sollen.

### Bedürfnisse, Erwartungen und Präferenzen hinsichtlich der medizinischen Versorgung in der ASV-Ambulanz

#### Bedürfnisse

Drei Patient:innen gaben an, dass ihre Bedürfnisse seitens der ASV-Ambulanz erfüllt worden sind. Dies führten sie auf die zufriedenstellende Patientenaufklärung, die erfolgreiche Schmerzbehandlung sowie die Versorgung mit den für sie entsprechenden Medikamenten und anderweitigen Therapieverordnungen zurück.

Drei Patient:innen hatten keine spezifischen Versorgungsbedürfnisse bezüglich ihrer Erkrankung geäußert. Dies begründeten sie mit ihrer bisherigen lästigen Krankheitsgeschichte, die ihnen die Hoffnungen und die Bedürfnisse hinsichtlich der Heilung bzw. der allgemeinen Versorgung ihrer Erkrankung nimmt. Eine Patientin äußerte, dass sie ihre Versorgungsbedürfnisse entsprechend an den verfügbaren Versorgungsmöglichkeiten der ASV-Ambulanz ausrichtet.

#### Erwartungen

Ein Großteil der Patient:innen (*n* = 7) berichtete, dass ihre Erwartungen hinsichtlich der Versorgung ihrer Erkrankung durch die ASV-Ambulanz erfüllt seien. Diese äußerten sie in folgenden Aspekten:

##### Alltagsbewältigung.

Zwei Patient:innen brachten zum Ausdruck, dass sie von der ASV-Ambulanz mehr Unterstützung bei der Bewältigung ihres Alltags erwarten. Sie berichteten von eingetretenen Einschränkungen der körperlichen Funktionsfähigkeit, die ihnen die Alltagsbewältigung erschweren.

##### Remission.

Zwei weitere Patient:innen äußerten die Erwartung an eine Remission, die das Therapieziel der rheumatologischen Versorgung darstellen soll.

##### Erhaltung des Status quo.

Fünf Patient:innen gaben zum Ausdruck, dass sie ihre Erwartungen ausschließlich auf die Erhaltung des Status quo und die Vermeidung einer Verschlechterung beschränken. Sie berichteten, dass die geringen Heilungschancen der chronischen Rheumaerkrankung ihre Erwartungshaltung beeinflussen.

##### Keine Erwartungen.

Eine Patientin äußerte ausdrücklich, dass sie keine Erwartungen an die ASV-Ambulanz habe, da sie von einem verbesserten Gesundheitszustand und Wohlbefinden berichtete und weitere Erwartungen hinsichtlich der Krankheitsversorgung nicht bestehen.

#### Präferenzen

##### Sachgerechte medizinische Versorgung.

Für eine kontinuierliche medizinische Versorgung ihrer Erkrankung äußerten die Patient:innen ihre Wünsche, dass sie weiterhin angemessene Verlaufskontrollen erhalten sowie mit Therapieverordnungen und Medikamenten und einer entsprechenden Medikamentenumstellung sowie -anpassung versorgt werden möchten.

##### Fachärztliche Weiterbildung.

Zusätzlich äußerten 2 Patient:innen folgenden Wunsch an die betreuenden Rheumatolog:innen, den sie bereits als erfüllt annehmen: Hierzu sprachen sie von der fachärztlichen Weiterbildung sowie Kompetenz- und Wissenserweiterung über den aktuellen Stand der Wissenschaft und Forschung in der Rheumatologie. Sie erhoffen sich hierdurch Informationen zu innovativen Behandlungsmethoden, die ihnen die vollkommene Heilung ihrer Erkrankung versprechen.

##### Patientenaufklärung.

Die Patient:innen äußerten ihre positive Erfahrung mit Rheumatolog:innen in der ASV-Ambulanz, die sie über bestehende Fragen zur Krankheitsbewältigung und Gesundheitserhaltung aufgeklärt haben. Sie präferieren die Fortsetzung der Art von Patientenaufklärung über diagnostische und therapeutische Maßnahmen. Zwei Patient:innen äußerten hierzu den Wunsch, zusätzliche Informationen zu neuen bzw. veränderten Therapiemöglichkeiten und der Wissenschaft sowie Forschung in der Rheumatologie zu erhalten.

##### Remission.

Zwei weitere Patient:innen äußerten ihren Wunsch, eine Remission zu erreichen, die das Hauptziel der rheumatologischen Krankheitsbehandlung ist.

##### Patientenwohl vor ökonomischem Druck.

Ein Patient äußerte den Wunsch an die Klinikleitung und die tätigen Rheumatolog:innen, den Fokus in der Versorgung ausschließlich auf das Patientenwohl zu beziehen und nicht auf das finanzielle Outcome. Hierzu ergänzte er, dass das Rheumazentrum baulich nicht erweitert werden soll, da die Erweiterung einen finanziellen Gewinn bringen würde. Zudem äußerte er die Empfehlung und seinen Wunsch, das Kliniksetting in seiner derzeitigen Struktur zu belassen, kontinuierlich an der Verbesserung der Versorgung zu arbeiten und eine Verschlechterung zu vermeiden. Das Patientenwohl soll weiterhin im Mittelpunkt stehen.

## Ergebnisse der Fokusgruppe – ÄiWB (t2)

### Arztwechsel.

Die FG-TN waren sich in ihrer Rückmeldung zu diesem Problem einig und begründeten den Arztwechsel damit, dass zum einen fachärztliche Weiterbildungen den Wechsel des Tätigkeitsbereiches erfordern und daher Rotationen zwischen ASV-Ambulanz und Station unvermeidbar sind. Zum anderen sind kurzfristige, mittelfristige und langfristige Faktoren wie Krankmeldungen (kurzfristig), Urlaub (mittelfristig) und Elternzeit sowie Stellenwechsel (langfristig) Gründe für den Arztwechsel. Laut FG-TN könnte mehr Personal diesem Problem entgegenwirken.

### Sprachbarriere.

Hinsichtlich der Sprachbarriere zeigten die FG-TN ihr Verständnis für diese Problematik und gaben an, der Sprachbarriere bemühend entgegenzuwirken. Trotz dieser Barriere bemühen sie sich, die Patientensicherheit durch explizite Aufforderung zur Nachfrage bei Unverständlichkeiten zu gewährleisten.

### Wartezeit.

Die FG-TN begründeten die Wartezeit mit akuten Notfällen und gaben ausdrücklich an, dass das Aufrufen der nächsten Patient:innen unmittelbar nach dem vorangegangenen Patienten geschieht, um Wartezeiten zu vermeiden. Die Minimierung der Wartezeit könnte durch die Einstellung von mehr Personal gelingen.

Für das Aufeinandertreffen von Arbeit und Termin äußerte eine Patientin den Wunsch, einen AU-Schein für die versäumten Arbeitsstunden ausgestellt zu bekommen. Laut FG-TN erfolgt für die gesessene Wartezeit keine Ausstellung eines AU-Scheins, sondern eine Bescheinigung für den Besuch der Klinik mit entsprechenden Zeitangaben, da es sich dabei um geplante, elektive Termine handelt.

### Terminmanagement.

Die FG-TN äußerten sich hierzu mit der Begründung, dass die Krankheitsaktivität die Häufigkeit der Terminierung bestimmt. Es steht für die ASV-Ambulanz außer Frage, dass krankheitsaktive Patient:innen mit erhöhten Entzündungswerten sofortige und zeitnahe Verlaufskontrollen erhalten und so häufig wie nötig gesehen werden. Patient:innen mit geringer Krankheitsaktivität bzw. Patient:innen, welche die Remission erreicht haben, werden zur halbjährigen Kontrolluntersuchung terminiert. Nichtsdestotrotz haben Remissionspatient:innen nicht weniger Anspruch auf medizinische Versorgung im Rahmen der ASV. Eine FG-TN verneinte die von dem Patienten zugeschriebene Ursache für die Terminierung. Es bleibt ausgeschlossen, dass Patient:innen aufgrund des Personal- und Terminmangels nicht bestellt werden. Die ASV-Ambulanz gewährleistet allen Patient:innen sachgerechte und professionelle medizinische Versorgung.

### Rezeptmanagement.

Hierfür wird die postalische Zustellung von Rezepten und Verordnungen gewünscht. Dieser Empfehlung stimmte eine FG-TN zu, wobei der rechtliche Rahmen dabei berücksichtigt werden muss.

### Patientenaufklärung.

Laut FG-TN beinhaltet die individuelle Patientenaufklärung als Minimum die wichtigsten und nötigsten Informationen über Diagnosestellung, Therapiemöglichkeiten und Medikamenteneinleitung. Eine tiefer gehende Patientenaufklärung erfordert zum einen mehr Behandlungszeit, und zum anderen erläuterte eine FG-TN, dass Patient:innen nach einer Aufklärung über Erkrankung, Therapie und Medikamente meist überfordert und nicht weiter aufnahmefähig seien, sodass noch eine zusätzliche Informationsvermittlung über die neusten Forschungsergebnisse und die Technologie an demselben Termin überkommend sei.

### Apparative Ausstattung.

Hierzu äußerte eine Patientin den Appell zur Anpassung der MRT-Ausstattung an krankheitsbedingte körperliche Deformitäten. Dieser Empfehlung stimmte ein FG-TN zu.

### Räumliche Orientierung.

Die Äußerungen einer Patientin zur Notwendigkeit an mehr Ausschilderung der Räumlichkeiten revidierten die FG-TN und verdeutlichten die Übersichtlichkeit des RZR und der ASV-Ambulanz.

### Verordnung und Rezepte.

Die FG-TN äußerten hierzu, dass die ASV-Ambulanz weiterhin chronisch erkrankte Patient:innen mit Verordnungen und Rezepten versorgen wird.

### Remission.

Die FG-TN merkten an, dass die Remission ein großer Patientenwunsch ist, der gleichermaßen auch das Ziel der Versorgung von rheumatologischen Erkrankungen ist. Alle FG-TN äußerten hinsichtlich der Behandlung von rheumatologischen Erkrankungen, dass sie zu den chronischen Krankheiten gehören und nicht vollständig geheilt werden können. Das Fortschreiten der Erkrankung wird durch Medikamente verhindert, auf diese die Patient:innen längerfristig angewiesen sein werden. Die Schmerzlinderung erfolgt in den meisten Fällen erst nach 12 Wochen.

### Handlungsempfehlung.

Die HE wurden durch die Forschungsdurchführende VI formuliert (Tab. 4).

**Tab. 4:** Handlungsempfehlungen zur Optimierung der Versorgungsqualität in der ASV auf Basis der Qualitätsdimensionen nach Donabedian

Aus den Patientenergebnissen und den Ergebnissen der FG wurden folgende HE abgeleitet:	
**Strukturqualität**	**Ergebnisqualität**
*SQ-HE 1: Arztwechsel* Personaleinstellung zur Regulierung des Arztwechsels	*EQ-HE: Behandlungserfolg* *Erhalt des Status quo, Vermeiden von Verschlechterung und Remission* Der Behandlungserfolg zeigt sich bei der Versorgung von rheumatologischen Erkrankungen in der Schmerzlinderung, Verbesserung der körperlichen Funktionsfähigkeit und Mobilität. Der Erhalt des aktuellen Gesundheitszustandes, das Vermeiden von Verschlechterung und Remission können als Aspekte der Ergebnisqualität gesehen werden. Alle Patient:innen merkten an, dass die Therapie zur Verbesserung der Krankheitsaktivität und Schmerzlinderung geführt hat. Zudem äußerten sie die Erwartung, den Status quo längerfristig zu erhalten und nicht mit einer Verschlechterung des Gesundheitszustandes konfrontiert zu werden. Die Remission ist ein großer Patientenwunsch, der gleicherweise auch der Qualitätsstandard (QS) der Versorgung von rheumatologischen Erkrankungen ist
*SQ-HE 2: Sprachbarriere* Betriebsinternes Briefing zur Arzt-Patient-Kommunikation	
*SQ-HE 3: Digitales Aufruf- und Nummernsystem* Einheitliche Strukturierung des Aufrufsystems – Anmeldenummer als Begleiter während des gesamten Behandlungsprozesses – Namentliches Aufrufen zum Labor – Patientenaufruf über ein Tonsignal (vorprogrammierte Stimme)	
*SQ-HE 4: Technische Funktionsfähigkeit* Die Gewährleistung der technischen Funktionsfähigkeit aller Monitore in den Wartebereichen ist erforderlich	
*SQ-HE 5: Apparative Ausstattung* Anpassung der MRT-Ausstattung an krankheitsbedingte Deformitäten. Dem ist hinzuzufügen, dass die Radiologie als selbstständiger Tätigkeitsbereich fungiert und kein Teil der RZR ist, wodurch seitens des RZR wenig Möglichkeiten bestehen, in die externe apparative Ausstattung der Radiologie mitzuwirken.	
*SQ-HE 6: Räumliche Orientierung* Mehr Ausschilderungen zur Sicherung der Orientierung und räumlichen Infrastruktur	
**Prozessqualität**	
*PQ-HE 1: Wartezeit* Personaleinstellung zur Wartezeitregulierung AU-Schein/Bescheinigung zur Entschuldigung der versäumten Arbeitszeit	
*PQ-HE 2: Terminmanagement* Regulierung der Terminierungen in entsprechender Absprache mit Patient:in	
*PQ-HE 3: Rezeptmanagement* Postalisches Zusenden der Rezepte und Verordnungen	
*PQ-HE 4: Medikamentenanwendung* Die zeit- und sachgerechte Medikamentenanwendung ist essenziell für die Krankheitsversorgung	
*PQ-HE 5: (Spezifische) Patientenaufklärung* Neben der individuellen Patientenaufklärung während der Behandlung, können betriebsinterne ambulante Patientenschulungen angeboten werden. Anhand der Patientenschulungen werden chronisch kranke Patient:innen über die Erkrankung, Therapien und den Umgang mit der Erkrankung geschult. Dieses Angebot besteht jedoch aktuell noch nur für stationäre Patient:innen	
*PQ-HE 6: Verordnungen und Rezepte* Kontinuierliche Versorgung mit Rezepten und Verordnungen (Physio‑, Ergo‑, Rheumakomplextherapie usw.) zur nachhaltigen Versorgung der Erkrankung	
*PQ-HE 7: Alltagsbewältigung* Unterstützung bei der Alltagsbewältigung seitens der ASV-Ambulanz	

## Diskussion

In dieser qualitativen Forschungsarbeit konnten Versorgungslücken mittels Patienteninterviews und einer FG aufgezeigt werden und basierend auf diesen Ergebnissen HE zur Optimierung der Versorgungsqualität in einem tertiären Zentrum formuliert werden.

Anhand der Patienteninterviews konnten die Zufriedenheit, Bedürfnisse und Erwartungen sowie die Präferenzen hinsichtlich der Versorgung identifiziert werden. Die gewonnenen Erkenntnisse zeigen eine Patientenzufriedenheit mit der ärztlichen Betreuung in der ASV und den in Anspruch genommenen medizinischen Behandlungsleistungen. Patienten*un*zufriedenheit ließ sich in folgenden Aspekten verzeichnen: Personalmanagement, interne (technische) Praxisausstattung, administrative Praxisabläufe sowie Behandlungsabläufe. Allgemein sprachen die Patient:innen von teils erfüllten Bedürfnissen und Erwartungen, die sich durch ihren verbesserten Gesundheitszustand, die ärztliche Unterstützung zur Krankheitsbewältigung sowie die Versorgung mit den für sie entsprechenden Medikamenten und anderweitigen Therapieverordnungen äußern.

Andererseits erwarten sie mehr Unterstützung bei der Alltagsbewältigung. Sie erwarten, den verbesserten Gesundheitszustand zu erhalten sowie eine Remission zu erreichen, und wünschen sich weiterhin eine sachgerechte medizinische Versorgung mit dem höchstmöglichen Behandlungserfolg – die Remission, eine spezifische Patientenaufklärung sowie die Weiterbildung von den behandelnden Ärzt:innen.

Die FG veranschaulichte eine kritische Reflexion der Patientenergebnisse, die ergab, dass die bereits aufgeführten Aspekte organisatorisch bedingt nicht vollständig behoben werden können, jedoch das Potenzial besteht, diese anhand entsprechender Maßnahmen zu optimieren.

In den hier abgeleiteten HE zur Optimierung der Versorgungsqualität geht es v. a. um die Stärkung der SQ und PQ, dessen Umsetzung auf die Steigerung der EQ abzielt. Das Umsetzungspotenzial lässt sich auf den ersten Blick bei dem Teil der HE, die einen geringeren Umsetzungsaufwand (wie beispielsweise technische Funktionsfähigkeit, räumliche Orientierung, Patientenaufklärung, Alltagsbewältigung) zeigen, eher verzeichnen als bei den HE, die hohe Anschaffungskosten (beispielsweise apparative Anpassung) und/oder das Umplanen unternehmensinterner Ressourcen und Abläufe erfordern. Zum Letzteren kann der Arztwechsel als ein Aspekt gesehen werden, der trotz Personaleinstellung nicht vollständig eingestellt werden kann, da abgesehen von zuvor genannten kurzfristigen, mittelfristigen und langfristigen Einflussfaktoren die Rotationen im Rahmen der fachärztlichen Weiterbildungen notwendig sind, um die rheumatologische Versorgung langfristig insgesamt zu sichern. Hierbei muss erwähnt werden, dass das Einhalten des Facharztstandards in der ASV-Ambulanz durch eine Besprechung aller Fälle mit einem Facharzt/Fachärztin (im RZR: Oberarzt/Oberärztin) gewährleistet werden muss.

Auch der Sprachbarriere könnte durch betriebsinternes Briefing nicht vollständig entgegengewirkt werden. Ein Briefing kann die Sensibilisierung und das Bewusstsein über bestehende sprachliche Barrieren stärken, doch nicht die Aufgabe eines Sprachkurses übernehmen. Der sprachliche Ausdruck kann, realistisch betrachtet, nicht kurzfristig verbessert werden. Um der Sprachbarriere entgegenzuwirken, sind weitere Maßnahmen notwendig.

Darüber hinaus lassen sich Wartezeiten als ein Teil der Prozessabläufe in der Gesundheitsversorgung sehen. Die Personaleinstellung kann zu einer Wartezeitverkürzung, jedoch nicht zur vollständigen Wartezeitvermeidung führen. Auch unter Betrachtung des Versorgungsalltages können Wartezeiten bedingt durch Notfälle nicht vollständig vermieden werden.

Die Umstrukturierung des digitalen Aufrufsystems weist ein Umsetzungspotenzial auf (beispielsweise Schaffung einer neuen Aufrufsystematik), doch fraglich ist, inwieweit im Rahmen von immer schärfer werdenden datenschutzrechtlichen Bestimmungen das namentliche Aufrufen der Patient:innen möglich ist.

Auch das postalische Zusenden von Rezepten und Verordnungen ist eine komfortable Lösung für immobile Patient:innen, doch verbunden ist dies zum einen mit zeit- und kostenintensiven Verwaltungsarbeiten und nicht weniger mit möglichen Versandrisiken und Risiken im Datenschutz.

Die Erfüllung der Bedürfnisse und Erwartungen der Patient:innen stellt im Rahmen der rheumatologischen Versorgung einen relevanten Aspekt dar. Die ASV-Ambulanz erfüllt die Bedürfnisse und Erwartungen der Patient:innen zum Teil. Hierzu konnte eine Perspektivenvielfalt der Patient:innen hinsichtlich der Erfüllung dieser verzeichnet werden. Diese Vielfalt kann sich anhand der subjektiven Wahrnehmung der Patientenkrankheitsgeschichte erklären lassen. Patient:innen mit einer langen lästigen Krankheitsgeschichte empfinden eine Hoffnungslosigkeit hinsichtlich einer Besserung ihres Gesundheitszustandes, diese ihnen die Äußerung ihrer Bedürfnisse, Erwartungen sowie Präferenzen hinsichtlich der Versorgung ihrer Erkrankung nimmt.

Es ist ersichtlich, dass die Repräsentativität der Ergebnisse aufgrund der 12 Patienteninterviews nicht so gegeben ist wie in dem Fall, in dem mehrere Hunderte Patienten befragt werden würden (wie es auch in großen Feld- oder klinischen Studien der Fall ist). Die Erkenntnisse aus den durchgeführten Interviews (*n* = 12) können nicht repräsentativ für alle Patient:innen in der ASV-Ambulanz gesehen werden, wenn man die Anzahl *n* = 12 im Verhältnis zur Gesamtpatientenanzahl betrachtet. Es ist jedoch wichtig anzumerken, dass die qualitative Forschungsarbeit die Entwicklung von Ansätzen zur Optimierung der Versorgungsqualität in der ASV-Ambulanz auf Basis der Patienteninterviews und der FG aufzeigt und somit darlegt, dass dieser Forschungsansatz für künftige Forschungen genutzt werden kann, um die Optimierung der Versorgungsqualität in anderen Krankenhaussettings zu untersuchen. Für das im Rahmen dieser Forschung betreffende Rheumazentrum empfiehlt sich eine weitere Forschung mit einer Erhöhung der Patientenstichprobe, um somit die Repräsentativität der Ergebnisse zu gewährleisten.

Zusammengefasst handelt es sich bei den abgeleiteten HE um erste wichtige Ansätze, die den aufgezeigten Optimierungsbedarf decken und zur kontinuierlichen Optimierung der Versorgungsqualität und nachhaltigen Qualitätssicherung in der ASV in einem tertiären rheumatologischen Zentrum dienen. Die nachhaltige und kontinuierliche Weiterentwicklung der Versorgungsqualität in einem solchen Setting ist von besonderer Bedeutung, die im Kontext dieser Forschung mit der Umsetzung dieser HE ermöglicht werden soll. Die Methodik und Ergebnisse dieser Studie können als Anhaltspunkt für Analysen anderer rheumatologischer Kliniken dienen, um im Rahmen des patientenorientierten Qualitätsmanagements und der kontinuierlichen Weiterentwicklung die Versorgungsqualität zu verbessern.

## Fazit für die Praxis

- Zur Sicherstellung der Nachhaltigkeit der patientenorientierten Versorgungsqualität in der ASV-Ambulanz sind kontinuierliche Überprüfungen der Versorgungsabläufe im Rahmen von Patienteninterviews und -befragungen essenziell.
- Im Rahmen weiterer Forschungen ist die Durchführung einer weiteren Fokusgruppe bestehend aus dem Pflegepersonal der ASV-Ambulanz sinnvoll, um ihre Perspektive hinsichtlich der Versorgung in der ASV-Ambulanz zu erheben.
- Um die Repräsentativität der Ergebnisse zu erhöhen, empfiehlt sich für eine erneute Studie die Kombination von quantitativen und qualitativen Forschungsmethoden („Mixed-Methods-Ansatz“). Darüber hinaus würde dieser Ansatz den Stichprobenumfang erhöhen und die Repräsentativität sowie die Qualität der Ergebnisse steigern.

## Abkürzungen

ASV: Ambulante spezialfachärztliche Versorgung
ÄiWB: Ärzt:innen in rheumatologischer Weiterbildung
EQ: Ergebnisqualität
FG: Fokusgruppe
FG-TN: Fokusgruppen-Teilnehmende
HE: Handlungsempfehlungen
PO: Patientenorientierung
PQ: Prozessqualität
QIA: Qualitative Inhaltsanalyse
RZR: Rheumazentrum Ruhrgebiet
